# SPASCER: spatial transcriptomics annotation at single-cell resolution

**DOI:** 10.1093/nar/gkac889

**Published:** 2022-10-16

**Authors:** Zhiwei Fan, Yangyang Luo, Huifen Lu, Tiangang Wang, YuZhou Feng, Weiling Zhao, Pora Kim, Xiaobo Zhou

**Affiliations:** West China School of Public Health and West China Fourth Hospital, Sichuan University, Chengdu 610041, China; Center for Computational Systems Medicine, School of Biomedical Informatics, The University of Texas Health Science Center at Houston, Houston, TX 77030, USA; West China Hospital, Sichuan University, Chengdu 610041, China; West China Hospital, Sichuan University, Chengdu 610041, China; School of Life Science and Technology, Xidian University, Xi’an 710126, China; West China Hospital, Sichuan University, Chengdu 610041, China; Center for Computational Systems Medicine, School of Biomedical Informatics, The University of Texas Health Science Center at Houston, Houston, TX 77030, USA; Center for Computational Systems Medicine, School of Biomedical Informatics, The University of Texas Health Science Center at Houston, Houston, TX 77030, USA; Center for Computational Systems Medicine, School of Biomedical Informatics, The University of Texas Health Science Center at Houston, Houston, TX 77030, USA; McGovern Medical School, The University of Texas Health Science Center at Houston, Houston, TX 77030, USA; School of Dentistry, The University of Texas Health Science Center at Houston, Houston, TX 77030, USA

## Abstract

In recent years, the explosive growth of spatial technologies has enabled the characterization of spatial heterogeneity of tissue architectures. Compared to traditional sequencing, spatial transcriptomics reserves the spatial information of each captured location and provides novel insights into diverse spatially related biological contexts. Even though two spatial transcriptomics databases exist, they provide limited analytical information. Information such as spatial heterogeneity of genes and cells, cell-cell communication activities in space, and the cell type compositions in the microenvironment are critical clues to unveil the mechanism of tumorigenesis and embryo differentiation. Therefore, we constructed a new spatial transcriptomics database, named SPASCER (https://ccsm.uth.edu/SPASCER), designed to help understand the heterogeneity of tissue organizations, region-specific microenvironment, and intercellular interactions across tissue architectures at multiple levels. SPASCER contains datasets from 43 studies, including 1082 sub-datasets from 16 organ types across four species. scRNA-seq was integrated to deconvolve/map spatial transcriptomics, and processed with spatial cell-cell interaction, gene pattern and pathway enrichment analysis. Cell–cell interactions and gene regulation network of scRNA-seq from matched spatial transcriptomics were performed as well. The application of SPASCER will provide new insights into tissue architecture and a solid foundation for the mechanistic understanding of many biological processes in healthy and diseased tissues.

## INTRODUCTION

Spatial transcriptomics (ST) has recently been developed to capture the spatial location of transcriptional activities within intact tissues (1–4), which cannot be achieved from traditional bulk transcriptome and single-cell RNA-sequencing (scRNA-Seq) data. Spatial transcriptomics has been applied to study the homeostasis/heterogeneity of tissue architectures in the brain (2,5–12), lung (13,14), breast (15–18), heart (19–21), liver (22–26), intestine (27–29), kidney (30–32), gastric (33,34), prostate (35,36), uterus (37), bladder (38), embryo (39–41), skin (42,43), etc. These studies have broadened our understanding of tissue organization at unprecedented molecular resolution in biomedical research, especially in developmental biology (9,29,44), regenerative medicine (21,27), disease/tumor microenvironment (2,15,16,30,35,38,42,45–52). Navarro *et al.* (11) found that detected spatially patterned genes in the mouse brain model could be used as potential molecular targets for the treatment of Alzheimer's disease. In the zebrafish tumor-microenvironment interface study (52), Hunter *et al.* identified several biological pathways, such as ‘lipid import into cell’ and ‘IMP biosynthetic process’, that were specifically distributed in the tumor microenvironment and associated with tumor growth and invasion. Current spatial transcriptomics techniques typically either adopt spatially-barcoded probes to locate and sequence mRNA abundance across tissue sections, known as next-generation sequencing-based (NGS-based) spatial transcriptomics (27,41,42,53–56) (i.e. 10X Genomics Visium, Slide-seq, HDST, DBiT-seq, etc.) or record the positions of mRNA profiles by multiple rounds of in situ hybridization, sequencing, and imaging, known as high-plex RNA imaging based (HPRI-based) spatial transcriptomics (4,57,58) (i.e. MERFISH, seqFISH, osmFISH, etc.). However, due to the limitations of transcriptome-wide coverage or sequencing depth of these spatial technologies, the current methods are still difficult to generate spatial transcriptomics where the transcriptome of each location can be reached to the unicellular level at large scales. Even though, with the ultrafast accumulation of publicly available scRNA-seq data, combining spatial transcriptomics data with matched specific contexts scRNA-seq can greatly improve the utilization efficiency of spatial data (59). Single-cell signatures can be used either as references to deconvolute NGS-based spatial transcriptomics data to predict cell-type proportions or map to HPRI-based spatial transcriptomics data to estimate cell-type distribution across tissue samples.

Currently, there are two representative resources of spatial data integration, SpatialDB (60) and STOmicsDB (61). STOmicsDB only provides a repository of literature and few datasets related to spatial transcriptomics topics, and does not provide any further analysis using these data. SpatialDB only conducts spatially variable (SV) genes analysis and enrichment analysis on only 10 primarily HPRI-based datasets (i.e. seqFISH and MERFISH). Due to the low transcriptome-wide coverage, HPRI-based datasets only contain hundreds of canonical genes (69–249), which were selected based on known biological knowledge. Besides, the results of each dataset are independent. It is challenging to explore their connections across different datasets. Most importantly, genes do not function alone; instead, they constantly interact with one another, and those biological interactions are critical for gene regulation, signal transduction, biochemical networks. Therefore, it is difficult to dig deep into the heterogeneity of tissue microenvironment compositions and their interactions based on the analysis from the current two existing spatial databases. Therefore, we developed a new database SPASCER, to systematically study the spatial heterogeneity of tissue organizations and their related biological processes at the unicellular level. SPASCER provides systematic annotations of spatial transcriptomics, including (i) spatially patterned genes, (ii) spatially patterned pathways, (iii) gene regulatory networks, (iv) cell–cell interactions and (v) spatial transcriptomics deconvolution and interactions.

In our database, we collected 1082 datasets from 43 studies across 16 tissue types and four species (human, mouse, chicken and zebrafish). Datasets included in our database are either HPRI-based or NGS-based spatial transcriptomics with corresponding scRNA-seq data. In total, we detected 12 116, 16 530, 1476 and 4915 unique spatial pattern genes for human, mouse, chicken and zebrafish, respectively. Through spatial pattern pathway analysis, we identified 22 792 pathways associated with 24 533 unique spatial pattern genes. The number of pathways identified from human, mouse, chicken and zebrafish was 7560, 7650, 7288 and 294, respectively. Gene regulatory network analysis was performed to detect cell-type specific key regulons and their downstream target genes, and 697 key regulons involved in 97 cell types were identified. We performed cell-cell interaction analysis using scRNA-seq to understand cellular behaviors and their responses to neighboring cells in the process of signaling transduction, and spatial transcriptomics data deconvolution analysis to estimate cell types distribution across tissue organizations. We also evaluated the spatial interactions based on the proximity of all of the co-expressing cells. The results of these analyses can be viewed from the online website (https://ccsm.uth.edu/SPASCER). We believe SPASCER will be a valuable reference resource for spatial transcriptomics analysis.

## MATERIALS AND METHODS

### Data collection

We searched the published literature on spatial transcriptomics from PubMed and released studies from bioRxiv using the keywords: ‘spatial transcriptomics’ OR ‘spatial transcriptomic’ OR ‘spatial genome’ OR ‘spatial RNA-seq’ OR ‘spatial sequencing’ (the query was done before May 2022). A total of 625 related papers were found. After selecting and pre-processing steps, all HPRI-based or NGS-based spatial transcriptomics data with matched scRNA-seq data were retained. Finally, we obtained data from 43 studies across 16 organ types and four species (Supplementary Table S1, Supplementary Figure S2A). We divided the dataset with multiple experimental replicates into different sub-datasets according to the given conditions. Therefore, 1082 sub-datasets were obtained. For the spatial transcriptomics data, spatial pattern analyses depend on the specific context of captured locations of each tissue sample. Besides, different tissue samples may have different coordinate systems due to different spatial technologies. For tissue samples using the same technology, the coordinate of captured spots may overlap, and integrating them may cause the loss of part spatial information. Currently, there exists one tool to integrate spatial transcriptomics data, PASTE (62), which is designed to align and integrate ST data from multiple adjacent tissue slices, and cannot be applied to different tissue types. Thus, for a study with several sub-datasets, we used Harmony (63) to remove batch effects and analyzed each sub-dataset separately. Most analyses were performed in R (version 4.1.2).

### Cell type annotation of scRNA-seq

To obtain spatial transcriptomics data at single cell resolution, scRNA-seq datasets are indispensable for our database construction. In order to annotate cell types of scRNA-seq data, raw counts were normalized using *SCTransform* firstly, then principal component analysis (PCA), clustering and Uniform Manifold Approximation and Projection (UMAP) dimensionality reduction were done using *RunPCA*, *FindClusters* and *RunUMAP* functions with default parameters from Seurat (64). Differential genes of each identified cluster were analyzed using *FindAllMarkers* function. Cell types of each cluster were inferred by comparing the top differentially expressed genes of each cluster with known cell-type canonical genes. The signature genes used to define each cell type are adopted from CellMarker database (65) and literatures, and can be found in Supplementary Table S2. Visualization and annotation of identified cell-types were displayed in the low dimension UMAP space. For the data with cell type annotation information, we used it directly for convenience. Data was processed in Seurat R package (version 4.1.0).

### Spatially patterned genes analysis

An important initial step in spatial transcriptomics analysis is identifying genes with highly spatial variation. Increasing evidence shows that the heterogeneity of those highly variable genes across tissue locations is closely associated with tissue organizations, cell states and microenvironment changes (8,11,19,43,66), providing opportunities to elucidate certain biological processes, and enhance our understanding of gene functions. Several computational methods (67–69) have been developed to estimate gene expression spatial variation by calculating the association between spatial coordinates and gene expression profiles. Unlike the trendsceek (67) and SpatialDE (68) used in SpatialDB, we adopted SPARK (69) for spatial pattern gene analysis. SPARK uses a generalized linear mixed model and different Gaussian kernels and periodic kernels to estimate spatial variation. The kernel matrices are computed automatically by coordinates of spots. SPARK yields well-calibrated *p*-values than trendsceek and SpatialDE. *CreateSPARKObject*, *spark.vc*, *spark.test* functions were used to create SPARK object, fit the statistical model, and test the spatially expressed pattern genes, respectively. Our analysis used raw count data and default parameters from SPARK (adjusted *P*-value < 0.05).

### spatially patterned pathway analysis

In addition to spatially patterned single genes, multigene-integrated pathways may also exhibit a high degree of spatial variation. As done in the melanoma study(52), we downloaded all human, mouse, chicken and zebrafish Gene Ontology (GO) terms from R package msigdbr (version 7.5.1) (70), and filtered out ‘biological process’ subontology for each species. There were 7568, 7656, 7655 and 7651 GO terms for human, mouse, chicken and zebrafish, respectively. For each GO term, the mean expression of associated genes involved in each spot was calculated across the tissue locations, then highly expressed spots were selected by defining spots whose mean expression value was above the 95% confidence interval. Euclidean distance was measured among these highly expressed spots. Next, an equal number of spots were randomly selected to compute the random distance, and this was repeated 1000 times to construct a null distribution. Finally, Wilcoxon's rank sum test was adopted to compute the *P*-value by mapping the true highly expressed distance onto the distance of the null distribution (*P*-value < 0.05), assuming that the aggregation of those highly expressed spots is likely to be in the active state of the metabolic pathways in the region.

### Gene regulatory network analysis

Gene regulatory networks can infer interactions among multiple genes (71,72). Transcriptional regulatory network (73) is designed to identify working transcription factors and related target genes. Analyzing gene regulatory networks can help to understand how gene modules are integrated to construct functional modules. We performed gene regulatory network analysis using scRNA-seq data, then checked whether those detected transcription factors and related target genes have spatial patterns or are involved in spatial pattern pathways. We conducted transcription factor regulatory analysis for each major cell type using pySCENIC (version 0.11.2) (73), a computational pipeline for transcription factor and gene regulatory network inference from scRNA-seq data. This tool infers co-expression modules between transcription factors and candidate target genes. Subsequently, regulons are obtained based on the enrichment of the transcription factor motif around the transcription start site of the potential target genes. pySCENIC uses *AUCell* to calculate AUC scores and rank the cells for a given regulon. In this study, we performed pySCENIC workflow using default parameters.

### Cell-cell interactions analysis using scRNA-seq

For multicellular organisms, cells interact with others by triggering downstream signal molecules through cognate receptors on the surface of the other cells, and it is vital for mediating diverse cellular functions, including immune responses, cellular differentiation and cell fate decisions (74). Cell–cell interactions were predicted based on scRNA-seq data by using CellPhoneDB software (version 1.1.0) (75). The average expression of each ligand–receptor pair was compared between different cell types, and only those with *P* < 0.05 were used for subsequent prediction of cell–cell communication.

### Spatial transcriptomics data deconvolution analysis

Spatial transcriptomics techniques, such as sci-Space, seqFISH and MERFISH, generated transcriptomics are measured in single-cell resolution; therefore, each spatial spot has been already at a single resolution, and each spatial spot has been labeled with a cell type. While spatial transcriptomics measured by NGS-based techniques, such as Visium, Slide-seq, HDST and DBiT-seq, contains several cells in one spot. Cell type prediction for each spot was calculated via the runPAGEEnrich function in Giotto (76). In this method, the cell-type specific marker genes were calculated from annotated scRNA-seq data that was provided by the source paper. Then the enrichment score was calculated based on the fold change of cell-type-specific marker genes for each spot.

### Cell–cell interactions analysis of Spatial Transcriptomics

Spatial cell–cell interactions were performed by Giotto toolbox as well. First, spatial spots were clustered using the *createNearestNetwork* and *doLeidenCluster* functions. Then a spatial grid with Delaunay neighborhood network was established by *createSpatialGrid* function. Cells located in a spatially proximal manner, as a proxy for potential cell–cell interactions were calculated by *cellProximityEnrichment* function. In order to find the spots involved in spatial interactions, *spatCellCellcom* function was used to calculate the communication score between the paired ligands and receptors for each spot. Significant ligand-receptors interactions were selected based on criteria of adjusted *P*-value < 0.05 and |log_2_FC| > 0.1.

### Drug and disease information

Disease and Drug information related to spatially patterned genes was extracted from DisGeNet (version7.0) (77) database and DrugBank (version 5.1.9) database (78), respectively. For each spatial pattern gene, we examined whether it is associated with known diseases and targetable by FDA-approved drugs.

### Database architecture

SPASCER is freely available at https://ccsm.uth.edu/SPASCER. SPASCER system is constructed based on a three-tier architecture: client, server and database. It includes a user-friendly web interface, a Perl's DBI module, and a MySQL database. This database was developed in MySQL 3.23 with the MyISAM storage engine.

## RESULTS

### Overview of SPASCER

**Figure 1.**
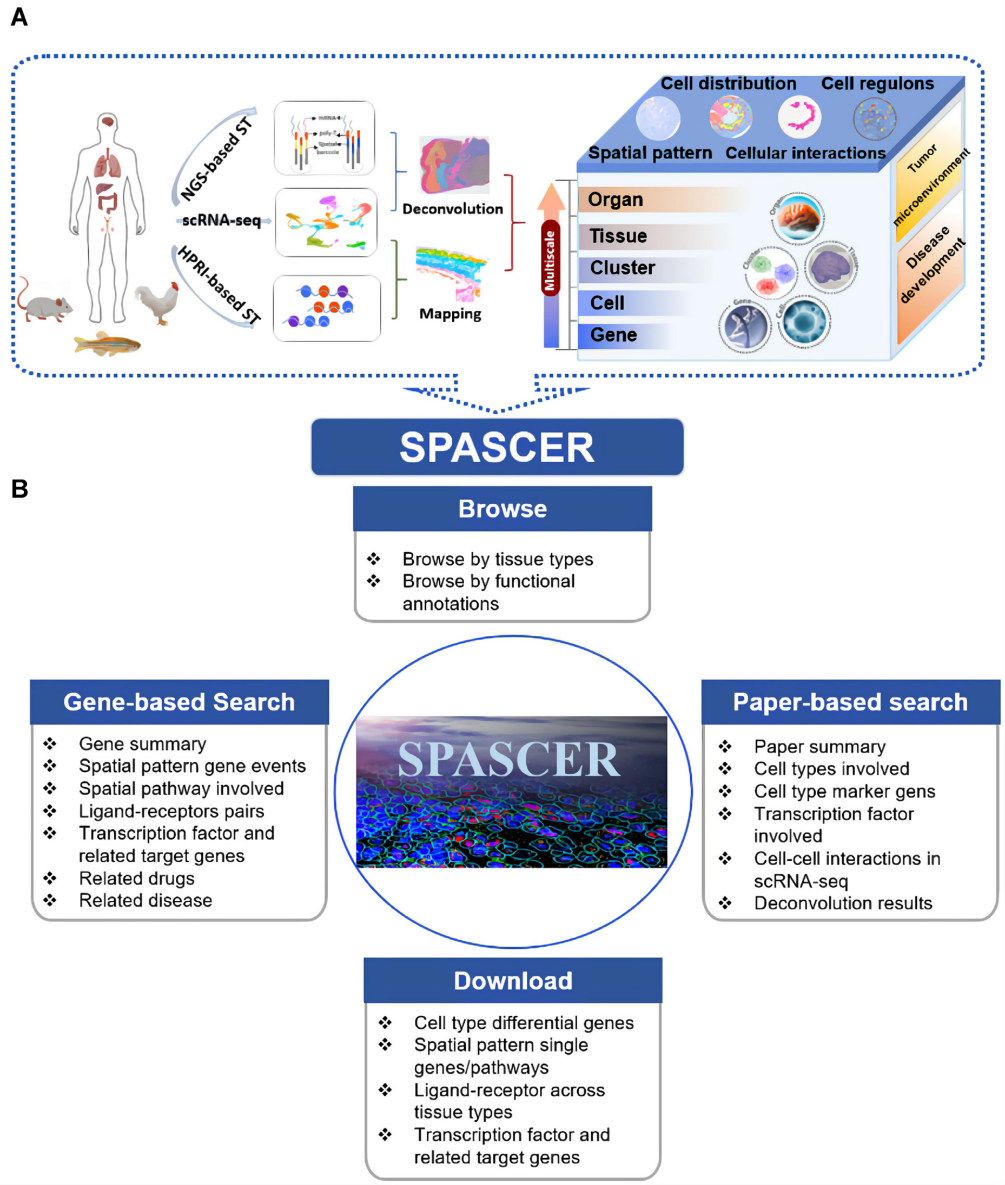
Overview of SPASCER. (**A**) SPASCER includes 43 datasets (1082 tissue samples) from 16 types of organs across four species (human, mouse, chicken and zebrafish). scRNA-seq data was used to map onto HPRI-based ST and to deconvolute NGS-based ST data, and through which cell type distribution could be estimated across tissue organizations. SPASCER provides spatial pattern gene analysis, spatial pattern pathway analysis, cell-cell interactions and gene regulatory network analysis, from the gene aspect to organ level. (**B**) Website interface of SPASCER. SPASCER provides gene-based and paper-based search. Users can query, browse and download related contents.

**Figure 2.**
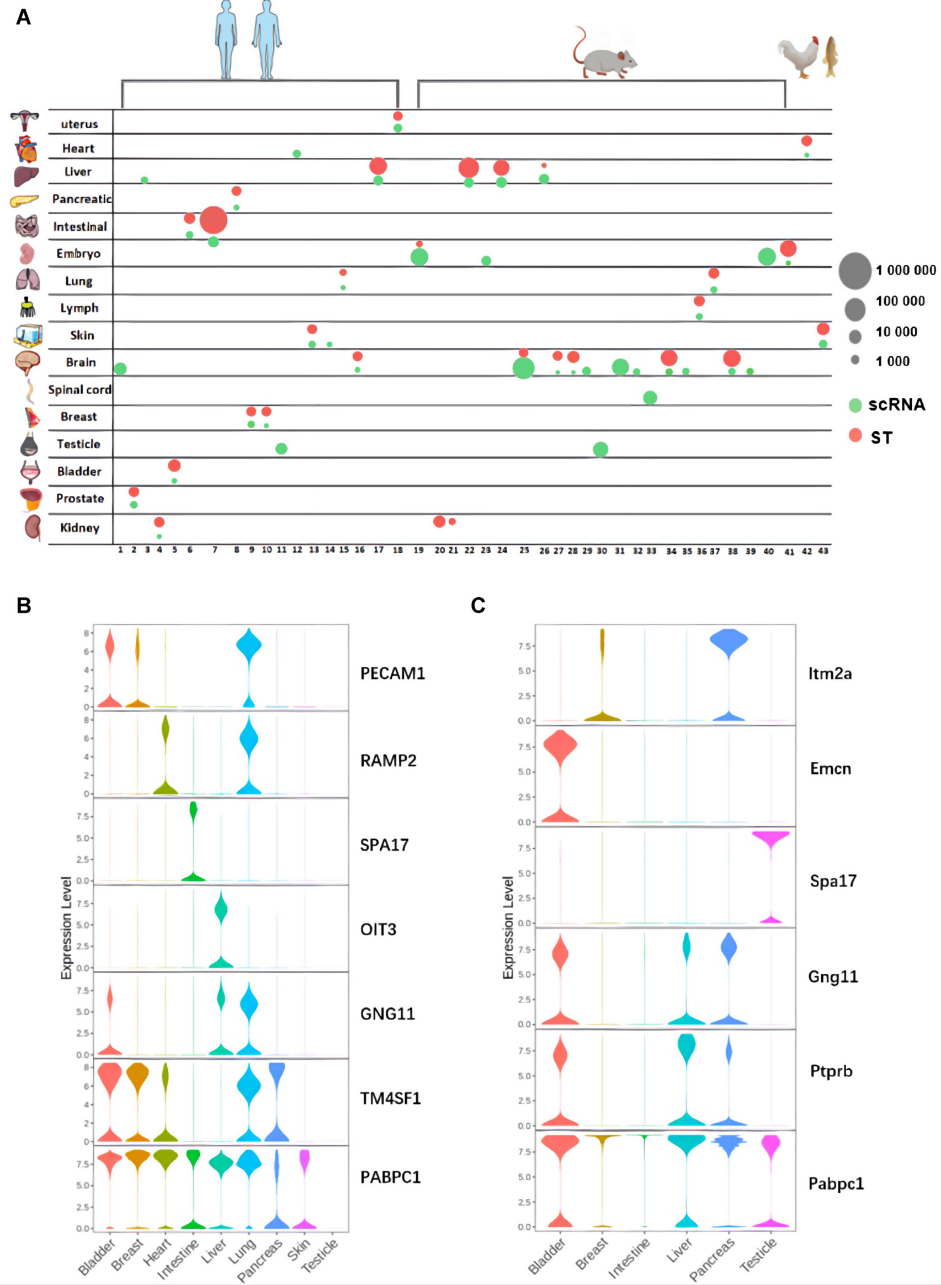
Summary of datasets used in SPASCER. (**A**) The number of cells from scRNA-seq (green colour) and spots from spatial transcriptomics data (red color) used in each data is indicated by the size of each circle. Brain contributed the largest number of papers. (**B** and **C**) Violin plots show expression levels of tissue specific endothelial marker genes across multiple tissues in human and mouse. PABPC1/Pabpc1 is highly expressed in multiple tissues both in human and mouse, while some, such as SPA17 and Emcn are highly tissue specific.

The design and construction of SPASCER is shown in Figure 1. SPASCER provided spatial transcriptomics data information in five respects (Supplementary Figure S1): spatially patterned genes, spatially patterned pathway, gene regulatory networks, cell-cell interactions and cell type deconvolution, aiming to characterize the heterogeneity of different organ tissues comprehensively. In total, we collected 1082 datasets from 43 studies which across 16 organ types and four species (human, mouse, chicken and zebrafish) (Figure 2A, Supplementary Table S1). A total of 118 cell types, including 263 minor cell types, were annotated. Cell types, such as endothelial, epithelial, fibroblasts, and immune cells are shared by multiple organs. For the spatially patterned genes, totally 35 037 unique spatially patterned genes were identified across all sub-datasets. Human, mouse, chicken and zebrafish had 12 216, 16 531, 1476, 4915 spatial heterogeneity genes, respectively. Combing available H&E-stained histological images, the specific distribution of those spatially patterned genes benefits us in looking into biological process at local scale, e.g. tumor region. For the Spatially patterned pathway analysis, we identified 22 792 pathways with spatial patterns across all the tissue samples, including 7560, 7650, 7288 and 294 pathways for human, mouse, chicken and zebrafish, respectively. Human breast, intestine, and uterus had the highest spatially patterned pathways numbers, which were 5922, 5313 and 4532, respectively. Gene regulatory network analysis identified 697 key regulons and a list of potential regulators, involving in a total of 97 cell types. Specifically, 541 transcription factors were identified in human tissue. *Jund* (jun D proto-oncogene) was the most detected regulon in mouse kidney, liver and lymph, involving in PT (proximal tubule segments) cell, monocyte, T cell, etc. Cell-cell interaction analysis using scRNA-seq identified 1020 unique ligand-receptor pairs in 135 cell types, while 1763 unique ligand–receptor pairs were detected in 97 cell types using spatial transcriptomics data.

**Figure 3.**
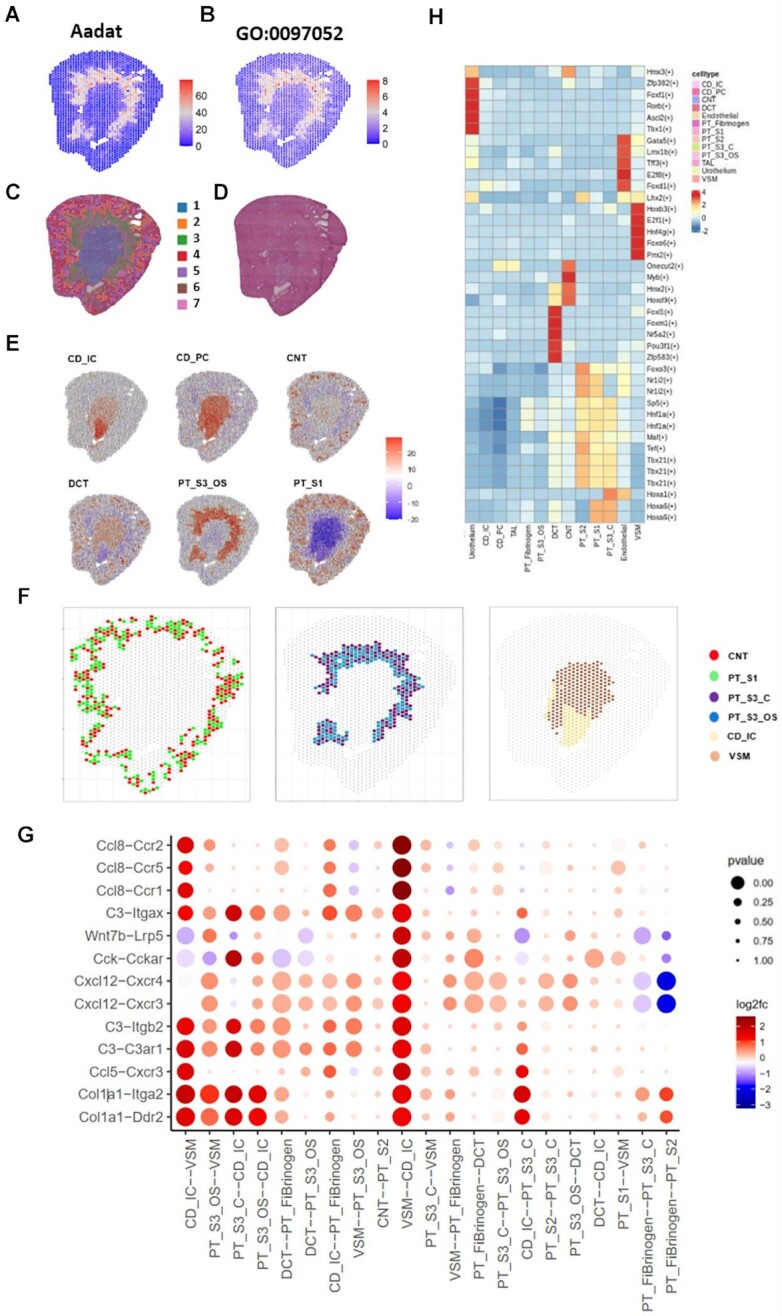
Example results of part functional annotations obtained from SPASCER. (**A**) SPASCER provides distribution map of spatial pattern gene that has highly spatial variation. Aadat is specifically distributed in the proximal tubule segments of kidney tissue. (**B**) Spatial pattern pathway across tissue architecture. GO:0097052, L-kynurenine metabolic process shows similar spatial pattern in the kidney proximal tubule segments, and has Aadat gene involved in the pathway. (**C**) Spots clustering shows tissue has clearly distinct layer. (**D**) Original H&E-stained histological images provides ground truth for comparison. (**E**) spatial transcriptomics deconvolution shows the probabilities of cell types distribution across tissue organization. (**F**) Cell-cell interactions using deconvoluted spatial transcriptomics shows highly enriched region pattern, and neighbouring cells have the same distribution or short distance are easier to interact with each other. (**G**) Selected significant ligand-receptor pairs for CD_IC cells and VSM cells, and among which Ccl8-Ccr2 has high interaction score. (H) Mast regulons detected in different cell types. Hoxb3, z2f1 and Foxo6 are specific TFs in VSM cells.

Combing with available H&E-stained histological images, the specific distribution of those spatially patterned genes and pathways would allow us to study biological processes at a local scale rather than whole tissue structures, e.g. tumor microenvironment. For the human squamous cell carcinoma data (42), *ITGB1* (integrin subunit beta 1) was specifically distributed in the boundary region between the tumor and stromal area. The spatial pattern of ITGB1 was identified in multiple other tissues, including bladder tumor, liver tumor, intestine, and breast tumor, consistent with the previous studies (79–81) showing that it could be pro-tumorigenic by enhancing cell migration and stromal invasion. We found that *LAMC2* (laminin subunit gamma 2) showed spatial pattern and was involved in spatial pathways such as ‘cell differentiation’ and ‘metabolic process’ in the tumor region, which was reported previously to promote tumor metastasis (82–84). Besides, *LAMC2*, *KRT8* and *KRT19* spatially patterned tumor markers shared ‘epidermis development’ and ‘cellular component morphogenesis’ spatially patterned pathways in the tumor region, which may indicate the proliferation of cancer cells. Except for the above tumor heterogeneity, detected spatial patterns may also reflect tissue organization and specific cell type distribution. For example, in the kidney mouse model, we found gene *Aadat* (aminoadipate aminotransferase) showed a clear spatial structure pattern (Figure 3A), and was involved in the spatially patterned pathway ‘L-kynurenine metabolic process’ (Figure 3B). Spatial spots clustering (Figure 3C) and H&E staining image (Figure 3D), and together with deconvolution analysis (Figure 3E) suggested that this specific spatial gene and pathway pattern might relate to the distribution of proximal tubule segments 3 cells (PT_S3_OS). The spatial pattern of *Aadat* was decreased after injury and increased with the repair process (Supplementary Figure S3B), which will help to study the regeneration process. As shown from the analysis above, we provide a rich resource of cell type marker genes, spatial gene patterns, spatial pathway patterns, gene regulatory networks, and cell-cell interactions of scRNA-seq and spatial transcriptomics. They are important clues to understand the generation of heterogeneity across tissues. Besides the examples we mentioned before, there are numerous genes or molecules that play a key role in biological processes. Researchers can download all the from our database for further study.

### Cell types category

Based on original cell type labels from single cell resolution spatial transcriptomics (MERFISH, seqFISH and osmFISH, sci-Space) and spatial deconvolution for NGS-based spatial transcriptomics (Visium, Slide-seq, HDST and DBiT-seq), 118 major cell types (263 minor cell types) were identified in total. Cell types, such as endothelial, epithelial, fibroblasts, and immune cells are shared by multiple organs, due to they are basic cell types in tissue construction. According to the statistical analysis of cell types, endothelial is the major cell type that is included in most studies (23/43) (Supplementary Figure S2B). Endothelial cells have important role in supporting the physiological activities of tissues. Their morphologies and functions are changed over time and location. In our database, nine human organs and six mouse organs included endothelial cells. The represent markers of endothelial cells across tissues are exhibited in Figure 2B and C. As shown in these figures, different tissues expressed unique marker genes, while polyadenylate-binding protein 1 (PABPC1, Pabpc1) is highly expressed in multiple tissues both in human and mouse (Figure 2B, C). Therefore, this could be a potential research point, as there are currently no reports revealing the relationship between polyadenylate-binding protein 1 and endothelial cells.

### Spatially patterned genes category

This category provides the genes that have spatial patterns. For the 1082 sub-datasets across 16 organ types, a total of 35 037 genes presented spatial heterogeneity across tissue architectures (Supplementary Figure S4A). In the website, we provided all the spatial pattern genes detected from each tissue type. For individual genes, we visualized the spatial distributions of all statistically significant samples. In this module, users can input gene symbols of interest to explore the spatial events for the genes. H&E-stained histological images are widely preferred for viewing tissue compositions by pathologists to help with disease diagnosis, especially for cancer. With the H&E image as the reference, we can select spatially patterned genes located in a specific region, such as the boundary between tumor and stroma. *ERBB2* (erb-b2 receptor tyrosine kinase 2), also known as *HER2*, showed spatial patterns in most breast cancer tissue samples, and particularly, was highly expressed in the tumor region, as reported to be overexpressed in ∼30% of human breast cancers (85). Besides, *ERBB2* also showed spatial patterns in liver and bladder tumor regions. The tumor-specific marker genes (i.e. *EPCAM*, *KRT8*, *EPCAM*, *B2M*, *FCA1* and *KRT19*) were also highly expressed in the tumor regions of prostate, colorectal, liver cancer, pancreas and breast cancer. The specific pattern may reveal the structure organizations in normal tissues also. *CCK* (cholecystokinin) was highly distributed in the L2, and L6 layers of human cortex, and *Aadat* was enriched in the proximal tubule region of mouse kidney. We also found that the spatial pattern rapidly decreased 2 hours after injury, almost disappeared in 2 days, and became normal after 6 weeks, which may relate to the injury and repairment process of the proximal tubule (Supplementary Figure S3B). Those detected spatially patterned genes will benefit our understanding of the tissue organization, and the dynamic changes of these pattern genes in time series tissue will broaden our knowledge of the key factors in tissue development, disease progression and injury regeneration. For different tissue types, the detected spatially patterned genes vary greatly, and for samples from the same tissue type, the spatially patterned genes also have some discrepancies. For example, in a human dorsolateral prefrontal cortex study, there are 12 sub-datasets, and the number of detected spatially patterned genes varies from 574 to 2337 (574, 579, 609, 620, 670, 713, 781, 861, 1557, 1583, 2108, 2337), with 246 genes consistent across all the sub-datasets. There are nine studies on mouse brain, with 325 sub-datasets, and the number of detected unique spatially patterned genes is 11 110, while there were no shared common spatially patterned genes for all those 325 sub-datasets, with only 2 genes (Cck, Mbp) shared by 324 sub-datasets. The reason may be that different regions of mouse brain, such as hippocampus, olfactory bulb, somatosensory cortex, etc. were analyzed. Besides, some studies used middle-aged adult mice, while some used embryonic mouse brain, and those situations are complexed. Tissue sample size could also affect the results. All the compared results could be found in Supplementary Table S3.

### Spatially patterned pathway category

This category provides the enriched biological pathways of the identified spatially patterned genes. In total, we identified 22 792 pathways with spatial patterns across tissue architectures using all samples (Supplementary Figure S4B). For the tumor studies, ‘cell migration’, ‘cell–cell adhesion’, ‘cell differentiation’, ‘cell proliferation’, ‘leukocyte migration’, etc., are highly enriched in tumor regions, indicating tumor proliferation and invasion. In the human intestinal development study (29), we found ‘epidermis development’, ‘epithelial tube morphogenesis’, ‘multicellular organism reproduction’, and other processes are highly enriched in the outer muscle. ‘Signal transduction by p53 class mediator’ was enriched in breast cancer with *CD44*, *CD73*, *CDH3* genes involved. For example, *LAMC2*, was involved in ‘cell differentiation’ and ‘metabolic process’ spatially patterned pathways in the human squamous cell carcinoma. In addition, we identified its spatial gene patterns that are located in the tumor area, which is consistent with previous reports that LAMC2 may promote tumor cell metastasis (82,84,86). We also found *LAMC2, KRT8* and *KRT19* spatially patterned tumor markers sharing ‘epidermis development’ and ‘cellular component morphogenesis’ spatially patterned pathways in the tumor region, which may indicate the proliferation of cancer cells. As shown in these examples, this annotation can help uncover the underlying biological functions of the spatial pattern genes.

### Transcription factor regulatory network category

This category provides the transcription factor regulatory network information. 697 key regulons and a list of potential target genes were identified, involving a total of 97 cell types. The detected potential regulons and target genes were mapped into protein–protein interactions to construct network. In this module, users can input the gene symbol of interest to check whether this gene plays a regulon role in multiple tissues, and also its potential target genes. For the previous study (paper)-based browsing, we provide all the identified significant transcription factors in each cell type using a heatmap (Figure 3H). In the kidney injury study (30), *Atf3* was identified as a transcription factor in the ischemia/reperfusion injury (IRI) and lipopolysaccharide mediated (LPS) mouse model. Atf3-targeting genes (*Tnfrsf12a*, *Spry1*, *Hck*, etc.) were highly enriched in the outer stripe in IRI model, and in the collecting duct in CLP model, suggesting that *Atf3* may be associated with neutrophil migration. In the human endometrium study (37), we found *HES1* is a key regulon in the early secretory, early-mid and mid proliferative periods during the menstrual cycle. Except for the endometrium, *HES1* was also estimated as a transcription factor in squamous cell carcinoma tissue, kidney IRI and Sham disease model, and lymph tissue, which has been reported as one of the downstream effectors of the Notch signaling pathway (87). As shown in this example, this category can provide the key regulons of the tissue architecture context.

### Cell–cell interaction category

This category provides the significant ligand-receptor pairs in the single-cell context. Ligand is a substance that forms a complex with a biomolecule released by one cell to signal either itself or a different cell. When a ligand binds to its respective receptor, the biological activity is altered to initiate several different types of cellular responses. Cell–cell interaction analysis using scRNA-seq data identified 1020 unique ligand-receptor pairs in 135 cell types. In the website, we provide all the identified significant ligand-receptor pairs and related source cells and target cells. For the gene-based search, the users can search for the individual genes’ interaction partners and expression levels in various cells. For the paper-based browse, the users can check all the significantly interacting cell types in a network module. Thicker edges indicate a stronger interaction. Cellular activities depend on cell–cell interactions, which are crucial for tissue homeostasis. Abnormal cell-cell interactions may lead to tissue disorder or even disease. For example, *MIF*-*CD74* ligand–receptor pair was most frequently identified in pDCs-cDC from melanoma tumors. Both *MIF* (macrophage migration inhibitory factor) and *CD74* (CD74 molecule) were found spatially expressed and distributed in many tumor tissues, including liver cancer, intestine injured tissue and pancreas. *MIF* was reported to play a role in the regulation of macrophage function in host defense (88) and was found involved in the spatially patterned pathways, such as ‘macrophage activation’, ‘macrophage migration’, ‘regulation of intrinsic apoptotic signaling pathway by P53 class mediator’, ‘regulation of signal transduction by P53 class mediator’, etc. Previous studies showed that *CD74* promoted breast and brain cancer metastasis (89–91) and was also found to be involved in the immune-related spatially patterned pathways, including ‘T cell differentiation’ and ‘leukocyte differentiation’ in our study. We infer that *MIF*-*CD74* may be a potential tumor therapeutic targets.

### Spatial deconvolution and interactions analysis category

This category provides highly enriched cellular neighborhoods and interactions through deconvolution analyses. Even though we made the prediction of cell type for each spot, in the complicated tissues, one spot may have a high enrichment score for more than one cell type. Therefore, we provide the enrichment heatmap of each dataset and analyzed the spot–spot communication based on Giotto clustering. Using annotated cell types from scRNA-seq, we estimated the distributions of individual cell types. Users can search whether they act as ligands or receptors across tissue architectures in different cells and can also search for estimated cell type distributions and whether they communicate with other cells. For example, in the kidney injury study, the spatial deconvolution analysis results showed that the proximal tubule segments 1 (PT_S1) and connecting tubule (CNT) were distributed in the outer layer, while collecting duct (CD) distributed in the inner region (Figure 3E). These results were consistent with the anatomical structures from H&E staining images. Specifically, we could estimate distinct cell-cell interaction patterns across the tissue architecture (Figure 3F). We found CNT cells highly interacted with PT_S1 in the outer layer, PT_fibrinogen interacted with PT_S3_OS in the middle region and VSM interacted with CD_IC cells in the inner region. Among the detected ligand-receptor pairs in CD_IC-VSM cells, *Ccl8* (C–C motif chemokine ligand 8) highly interacted with *Ccr1* (C–C motif chemokine receptor 1), *Ccr2* (C–C motif chemokine receptor 2), *Ccr5* (C-C motif chemokine receptor 5) (Figure 3G), and these may due to the kidney injury process, since previous studies showed these chemokine genes family have a relation with inflammation and macrophage associated metastasis (92–94). As mentioned above, these region-specific cell type distribution and interactions would help us understand the potential mechanism in the disease microenvironment.

## DISCUSSION

SPASCER is a unique database that systematically annotates spatial transcriptomics data at single-cell resolution from four species. Studying spatial transcriptomics at the single-cell level contributes to a more comprehensive understanding of the heterogeneity of tissue organizations and related biological processes. Spatial pattern analyses, including spatial single gene patterns and spatial pathway patterns, allow us to explore highly spatially variable genes/pathways across physical locations and focus on specific regions, such as the tumor interface. Through SPASCER, the users also can explore tintercellular interactions using the expression profiling of ligands and receptors in the tumor/disease microenvironment. These insensitive annotations will facilitate the discovery of cell states and the detection of potential therapeutic targets. Even though spatial transcriptomics has been widely used in biomedical studies, the available data for certain organs is still limited, specifically time series data. However, with the rapid development of ST sequencing technology, high-resolution platforms for spatial multi-omics data are becoming accessible, including proteomics and ATAC. We will continue to integrate more valid spatial multi-omics datasets and extend SPASCER into organ-based atlases (even 3D based-organ atlases) to gain a more comprehensive understanding of tissue heterogeneity. In order to keep SPASCER as the forefront of the spatial transcriptomics database, new data will continuously be collected and updated to our database every 6 months, and users could also share publicly available spatial transcriptomics data on our website. Furthermore, the usage of H&E images falls far short of the information they contain. We will employ deep learning-based transformation methods to extract useful information from them, and integrate them with gene expression profiles to extend the level of understanding from simple tissues to more complex structures. We believe that SPASCER will provide new insights into tissue architecture and a solid foundation for the mechanistic understanding of many biological processes in healthy and diseased tissues.

## Supplementary Material

gkac889_Supplemental_Files
