# Supplementary material for: SPASCER: spatial transcriptomics annotation at single-cell resolution
Source: Nucleic Acids Res. 2022 Oct 16;51(D1):D1138–49. doi: 10.1093/nar/gkac889 (PMC9825565; doi:10.1093/nar/gkac889)
Supplement: gkac889_Supplemental_Files [file gkac889_supplemental_files.zip › Legends of supplementary table 1-3.docx]

**Legends of supplementary table S1-S3**

**S1:** Detailed information of each study used in SPASCER

**S2:** Signature genes of different cell types in tissues

**S3:** Consistent spatially patterned genes across tissue samples
