# Supplementary material for: SPASCER: spatial transcriptomics annotation at single-cell resolution
Source: Nucleic Acids Res. 2022 Oct 16;51(D1):D1138–49. doi: 10.1093/nar/gkac889 (PMC9825565; doi:10.1093/nar/gkac889)
Supplement: gkac889_Supplemental_Files [file gkac889_supplemental_files.zip › Supplementary_figures.pdf]

SPASCER: SPAtial transcriptomics annotation at Single-CEll Resolution

Zhiwei Fan[1,2,*,†], Yangyang Luo[3,†], Huifen Lu[3,†], Tiangang Wang[4], YuZhou Feng[3], Weiling Zhao[2], Pora Kim[2,*], and Xiaobo Zhou[2,5,6,*]

[1]West China School of Public Health and West China Fourth Hospital, Sichuan University, Chengdu 610041, China
[2]Center for Computational Systems Medicine, School of Biomedical Informatics, The University of Texas Health Science Center at Houston, Houston, TX 77030
[3]West China Hospital, Sichuan University, Chengdu 610041, China
[4]School of Life Science and Technology, Xidian University, Xi'an 710126, China
[5]McGovern Medical School, The University of Texas Health Science Center at Houston, Houston, TX 77030, USA
[6]School of Dentistry, The University of Texas Health Science Center at Houston, Houston, TX 77030, USA

*Address correspondence to:
Xiaobo Zhou, Ph.D. and Pora Kim, Ph.D.
School of Biomedical Informatics
The University of Texas Health Science Center at Houston
7000 Fannin St., Houston, TX 77030
Phone: 713-500-3923 and 3636
Email: Xiaobo.Zhou@uth.tmc.edu and Pora.Kim@uth.tmc.edu

[†]These authors contributed equally to this work.

Supplementary Figures

A.

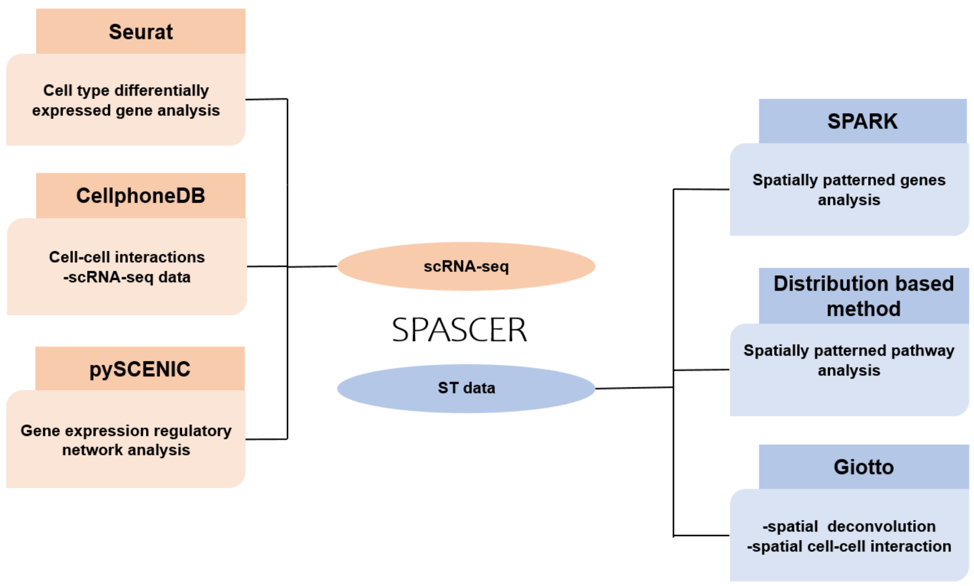

Supplementary Figure 1 A. Feature functions we performed on scRNA-seq data and ST data. SPASCER provides systematic annotations of spatial transcriptomics, including 1) spatially patterned genes, 2) spatially patterned pathways, 3) gene regulatory networks, 4) cell-cell interactions, and 5) spatial transcriptomics deconvolution and interactions.

A.

Organ	Num of papers	Num of tissues
brain	13	412
embryo	4	38
intestine	3	20
liver	3	28
kidney	3	13
skin	3	21
breast	2	42
lung	2	6
lymphoid	2	14
bladder	1	4
heart	2	23
pancreas	1	10
prostate	1	12
spinal cord	1	429
testicle	1	6
uterus	1	4

B.

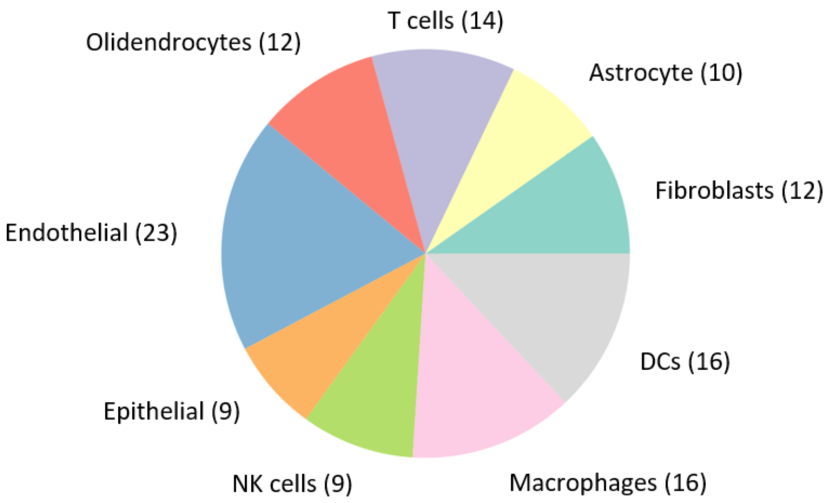

Supplementary Figure 2. A. Summary of the tissue samples used for each organ. **B.** The top 9 major cell types that shared by multiple studies. Cells come from those studies can be classified in to 118 major cell types or 263 minor cell types. Some of them are shared by multiple tissues, above picture showed the top 9 major cell types included in multiple studies, the number of studies exhibited in the bracket.

A.

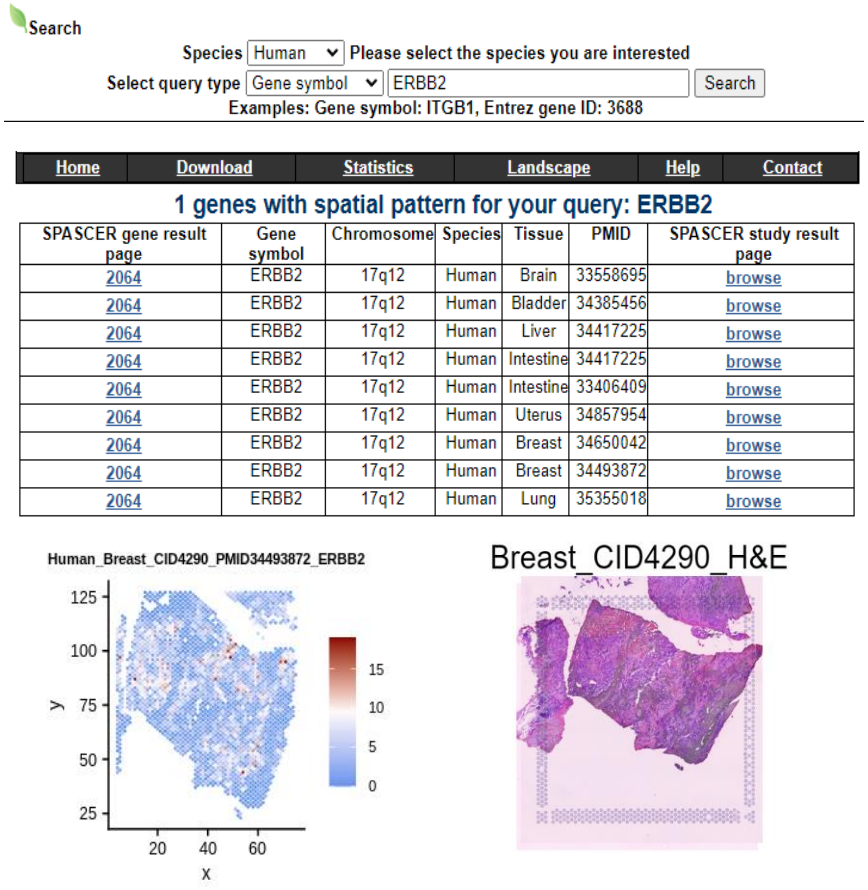

SPASCER gene result page	Gene symbol	Chromosome	Species	Tissue	PMID	SPASCER study result page
2064	ERBB2	17q12	Human	Brain	33558695	browse
2064	ERBB2	17q12	Human	Bladder	34385456	browse
2064	ERBB2	17q12	Human	Liver	34417225	browse
2064	ERBB2	17q12	Human	Intestine	34417225	browse
2064	ERBB2	17q12	Human	Intestine	33406409	browse
2064	ERBB2	17q12	Human	Uterus	34857954	browse
2064	ERBB2	17q12	Human	Breast	34650042	browse
2064	ERBB2	17q12	Human	Breast	34493872	browse
2064	ERBB2	17q12	Human	Lung	35355018	browse

B.

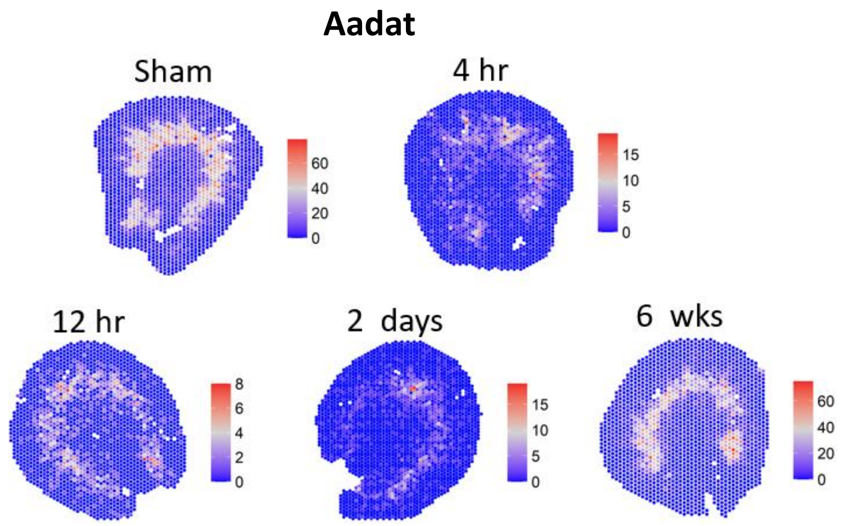

Supplementary Figure 3. A. Example results of spatially gene pattern analysis of ERBB2 gene. **B.** Time series pattern of Aadat in mouse kidney. the spatial pattern of Aadat was rapidly decreased in 2 hours after injury, almost disappeared in 2 days, and became to normal after 6 weeks, which may relate with the injury and repairment process of proximal tubule.

A.

	Chicken	Human	Mouse	Zebrafish
**Bladder**	0	1305	0	0
**Brain**	0	2810	10666	0
**Breast**	0	333	0	0
**Embryo**	0	0	3920	0
**Heart**	1476	211	0	0
**Intestine**	0	702	5084	0
**Kidney**	0	0	7470	0
**Liver**	0	5441	1943	0
**Lung**	0	187	4247	0
**Lymph**	0	0	5466	0
**Pancreas**	0	40	0	0
**Prostate**	0	399	0	0
**Skin**	0	164	0	4915
**Spinal cord**	0	0	1258	0
**Testicle**	0	0	5722	0
**Uterus**	0	624	0	0

B.

	Chicken	Human	Mouse	Zebrafish
**Bladder**	0	705	0	0
**Brain**	0	258	7472	0
**Breast**	0	5922	0	0
**Embryo**	0	0	6618	0
**Heart**	7288	0	0	0
**Intestine**	0	5218	678	0
**Kidney**	0	0	5747	0
**Liver**	0	150	5460	0
**Lung**	0	511	504	0
**Lymph**	0	0	4463	0
**Pancreas**	0	1109	0	0
**Prostate**	0	0	0	0
**Skin**	0	2993	0	294
**Spinal cord**	0	0	6417	0
**Testicle**	0	0	0	0
**Uterus**	0	4532	0	0

Supplementary Figure 4. A. Summary of identified spatially patterned genes. **B.** Summary of identified spatially patterned pathways.
